# Shifting landscapes of digital literacy

**DOI:** 10.1007/s44020-022-00019-x

**Published:** 2022-08-23

**Authors:** Alexander Bacalja, Catherine Beavis, Annemaree O’Brien

**Affiliations:** 1 The Melbourne Graduate School of Education, The University of Melbourne, Parkville, Australia; 2 Office of the Executive Dean, Arts and Education, Deakin University, Melbourne, Australia

**Keywords:** Digital literacy education, Critical digital literacies, Digital citizenship, Pedagogy, Data literacies

## Abstract

This paper explores how changing digital literacy practices in educational contexts require that we continually revisit conceptualisations of digital literacy education. We begin by analysing the positions taken by stakeholders who contribute to digital literacy discourses in Australia, exploring how competing interests produce effects which manifest in ways that differently consecrate social and cultural practice in the digital age. We advocate the need for pedagogic frameworks that support digital literacy education. Existing approaches tend to privilege the operationalisation of digital technology. By contrast, teaching is needed which focusses on meaning-making and creating. However, the ‘datafication of everyday life’ (Barassi, 2018, p.170) has included extraordinary interventions into schooling that have significant implications for teachers and students. We argue that preparing young people for digital citizenship must include a focus on critical digital literacies that are responsive to contemporary digital forces (e.g. platformatisation, artificial intelligence, edu-apps, algorithms) as well as those digital technologies that are yet to make their way into formal schooling.

## Digital literacy

It has become a truism to observe that the world has been radically changed by the digital and to note the pervasiveness of digital platforms and technologies in everyday lives, and the effects of these technologies on users. Prior to COVID-19, the importance of digital literacy, and the need for young people to become effective and agential users and makers of digital forms, texts and practices was already widely recognised (Knobel & Lanskear, 2008; Jenkins, 2009), and embedded in state and national curricula (e.g. Victorian Curriculum and Assessment Authority, 2016; ACARA, n.d.). With the advent of COVID-19, the need for digital literacy has been thrown into high relief (Gourlay et al., 2021; Karagul, Seker and Aykut, 2021). As Karagul, Seker and Aykut (2021) put it ‘The abrupt transition from face-to-face to online education has created the need for some specific abilities, such as digital literacy on the side of the learners at all educational levels’ (p.1).

A great deal has been achieved in identifying key features of multimodal literacy, from which semiotic accounts of digital literacy grew. Perhaps the most influential of these was the concept of literacy as design (Kress & van Leeuwen, 2006). Literacy as design entails ‘the transformative use of available representational resources in the production of new meaning’ (Lam, 2000, p.1193) including linguistic, visual, audio, gestural, spatial and multimodal modes, with multimodal ‘the most significant as it relates all the other modes in quite remarkably dynamic relationships’ (New London Group, 1996, p.80). Within these frames, meaning making is understood as being situated and socially constructed, encompassing a wide range of semiotic elements and resources (Gee, 1996). This way of thinking about literacies creates space for the conceptualisation of digital literacy (Bawden, 2008; Buckingham, 2006; Bulfin & McGraw, 2015), recognising the ubiquity of the digital in everyday life and the need to prepare young people for a lifetime of digital practice. Yet as Pangrazio (2016) notes, ‘[d]efining what is meant by digital literacy has proven complicated as the spaces, texts and tools which contextualise such practices are constantly changing.’ (p.163). She points to distinctions between broad definitions whereby digital literacies are defined as ‘semiotic activity mediated by electronic media’ (Thorne, 2013, p.192) through to more narrowly focussed definitions, emphasising on the one hand mastery and operational proficiency, and on the other, evaluation, creativity, and critique (Lankshear & Knobel, 2011).

Our concern with the composition and enactment of digital literacy education can be captured by questions such as the following: what do we want young people to know and be able to do with digital technologies? What does the curriculum say about the knowledge and skills that underpin such an education? How prepared are teachers to support this work? What do students already know about the digital world and how are they navigating digital spaces? The challenge of determining what exactly constitutes a ‘good enough’ digital literacy education is further complicated by the stratification of the field into sub-fields of inquiry. Much like Gee’s (1996) assertion that we never read generally, but are always reading specifically, digital literacy practices are always situated within specific discourse communities, and specific in nature; be they platform literacies (Williamson, 2017a, 2017b), digital game literacies (Bourgonjon, 2014), social media literacies (Pangrazio & Cardozo Gaibisso, 2020), data literacies (Pangrazio & Selwyn, 2019), media literacies (Burn & Durran, 2007), consumer literacies (Elms et al., 2016), and many others. Do we expect students to finish their schooling as masters of each of these domains, or do we need broad concepts that can be applied across the fields?

In this paper, we explore how digital literacy education might be reconceptualised so as to represent a constantly evolving body of knowledge and practice that is responsive to the changing digital literacy landscape. We begin by analysing the positions taken by stakeholders who contribute to digital literacy discourses in Australia, exploring ways in which competing interests produce effects which manifest into practices that differently consecrate social and cultural practice in the digital age. We argue conceptual thinking about what constitutes digital literacy must be broadened beyond the imperatives of operationalising digital technologies, and engage rather with understandings and practices of contemporary digital literacies. Through a focus on two examples of such literacies, platformalisation and edu-apps, we hope to show how preparing young people for digital citizenship must be constantly responsive to the critical imperatives of contemporary digital forces.

## Stakeholder positions and digital literacy discourse

The pathway forward for literacy educators seeking to engage in digital literacy education is characterised by conceptual multiplicity. While curricula go some way to outlining the desirable knowledge, skills, and dispositions we hope learners will take from their time in primary and secondary education, the literacy landscape has created a context whereby teachers are facing push and pull factors that are almost impossible to reconcile.

Analysing the positions taken by those stakeholders contributing to digital literacies’ discourse in Australia reveals some of the conceptual multiplicity within which teachers are expected to work. These positions, advocated by stakeholders including the state, industry groups, academics, and the media, are neither ideologically neutral nor coherently and consistently communicated. Each represents an interest in the field of literacy education. In the Bourdieusian tradition of mapping the positions in the field which shape the field (Bourdieu, 1993), examining how these interests — themselves a product of history, and, simultaneously, through their continued reproduction, producers of history — compete and struggle with each other over influence of the ‘truth’ of the field, may uncover pathways forward.

The state, including the government officials charged with creating and enacting the policies of elected representatives, has presented confusing and contradictory messages regarding the relationship between young people and the literate practices, digital and non-digital, that are communicated as important for school and post-schooling success. On the one hand, digital literacy is subordinated to more important ‘basic’ literacy. Through decades of state and nationally mandated high-stakes literacy testing, coupled with an indefatigable ‘back to basics’ discourse, what counts as literacy is what can be tested, and what can be tested is what counts. As the Minister for Education recently claimed, to improve literacy standards, we must, ‘de-clutter their curriculums and get back to basics’ (Tehan, 2019). It is towards this goal that schools are told they must work, prioritising decontextualised operational functions that deprofessionalise the teacher, reducing their autonomy, while simultaneously subjecting them to increasing regimes of accountability. The constant emphasis from political leaders on school performance on these testing regimes legitimises narrow conceptualisations of literacy, and reasserts the state’s authority over all other actors, an example of states’ execution of what Bourdieu calls their prerogative as the ultimate arbiters of acts of consecration (1998, p. 51), and determining what counts and who will enforce what counts.

At the same time, teachers and schools are expected to respond to the rapidly changing technological environment and create space for the development of knowledge and skills associated with information and communication technologies. Government programmes that deploy computers into every school across the nation, coupled with a rhetoric which ties digital competencies with national economic interests (Council of Australian Governments Education Council, 2019), have been supported by state and national curricula, which suggest these new skills and knowledge should be embedded into every discipline area. Ignoring the long history of organising schooling according to strict discipline areas (Yates et al., 2017; Young, 1998), this has required teachers to ‘square the circle’, finding places in highly specialised subjects areas to also do the work of the digital curriculum. The ‘basics’ which so often dominate government discussions around literacy now appear insufficient for a future of work.

Industry groups interested in the skills and knowledge of graduates who enter the workforce have also presented contradictory positions regarding desirable literacy outcomes, including digital literacy skills and competences. On the one hand, the reports produced by these groups reference standardised testing to establish the under-performance of Australian students in terms of literary testing, especially in contrast to other OECD nations (Australian Industry Group, 2016). The conclusions they draw suggest that this is negatively impacting Australia’s international competitiveness. In doing so, they further legitimise this narrow and highly contested notion of literacy, and, through their interactions with government, place further pressure on teachers to spend more energy in this area.

On the other hand, reports also advocate for graduates with more diverse and complex digital literacy-orientated capabilities, with an emphasis on communication and creativity (NSW Business Chamber, 2017). Work is prioritised as the most important outcome of schooling, and successful workers are those who possess a range of flexible and adaptable digital literacies. However, there is also an emphasis on developing digital literacy skills for life beyond work. The importance of supporting young people to promote and demonstrate ethical, productive, and socially cohesive uses of digital technology is tied to the responsibilities of good citizens (NSW Business Chamber, 2017). Digital skills, it is argued, provide access to full participation in modern life, including giving adults access to many basic services. Digital skills, in the context of young people, should also include the capacity for them to understand their digital presence and how they consume online (NSW Business Chamber, 2017, p. 28).

An alternative and diverse series of positions are taken by the many academics and literacy theorists interested in school-based literacy instruction. The emergence of new conceptualisations of literacy, such as new literacies, critical literacies, media literacies, digital literacies, and multiliteracies, challenges the notion of literacy as a singular concept. Teachers are left to determine which approach best suits their context, and whether they can do justice to those they cannot authentically address.

Those working in initial teacher education have significant influence over which of these positions, and their associated ways of thinking and doing, graduates will take with them to placement and post-training teaching. Novel approaches to twenty-first century forms of literate practices are increasingly being theorised in terms of pedagogical interventions, producing innovative practices that conflict with school contexts that privilege maximising measurable student-achievement. The tensions must ultimately be resolved by the teachers themselves, as they are expected to balance the pressure for local measurable ‘basic’ literacy improvements with the goals of ‘new literacies’ thinking. At times technology is presented as a panacea for the challenges of contemporary schooling (for example, supporting over-worked teachers through new software, minimising geographic challenges, and improving youth mental health via ‘smart apps’). At other times technology is seen as the cause of all the social ills of today’s youth. A two-dimensional view of youth interactions with and through digital technologies has continued throughout the pandemic, with digital technologies lauded for their ability to keep students learning during times of school closures, but also criticised for distracting young people from more worthwhile pursuits.

Framed within a mythologisation of a golden age not hindered by ‘all-consuming’ digital technologies, a discourse emerges which holds digital devices, the Internet, and video games as obstacles to the growth of a generation ready for the literacy challenges of life. The confusion regarding the relationship between digital practices and literacy is understandable given the public has been positioned to think about ‘the literacy crisis’ in terms of digital technologies as both a cause, and simultaneously the tool to mitigate the crisis.

Each of the above positions represents a stake in the game. We refer here to Bourdieu’s metaphorical use of ‘the game’, which he draws on to examine the games of culture which are inherently tied to education systems and their imposition of cultural practices (1993). These practices matter greatly as they become internalised as dispositions for future action. The different stakeholders discussed above, as well as many others that space does not allow us to explore here, bring with them a specific interest in the game, implied by their participation. This interest, *illusio*, captures their investment in the game and is tied to beliefs about the field and the investments which should produce distinction in such a field. Digital literacy education has come to represent many forms of knowledge, skills and practices because of the distance between those positions discussed above, as they struggle to consecrate particular cultural dispositions.

Our concern is that despite the incongruent nature of many of these positions, there is an expectation that literacy educators can navigate this landscape unproblematically with their students. This expectation ignores what sociology has taught us about the origins of people’s dispositions and their ability to perceive the field as a field of possibilities. All dispositions are associated with certain social and cultural origins (Bourdieu, 1993, p. 70), and the construction of digital literacy education as a space for multiple practices is no different.

Adding to the challenge is the incorporation of digital technologies into schools at such a rapid rate that digital literacy education is now bound to a range of new sociomaterial entanglements. The rise of digital literacies associated with datafication, AI, platformatisation, algorhythmic learning, and big data, to name a few, raises further questions about the very nature of digital literacy in schools, what should constitute a digital literacy education, and what role teachers should have in preparing young people for a future digital citizenship.

## A shifting conceptual landscape

There has been evolution of conceptual thinking in terms of how educational institutions might address digital literacy education. Early interest in digital literacies tended to focus on the provision of computers and the skills necessary to operationalise this technology (Molnar, 1978). As iterations of digital technology have emerged, the emphasis on productive capacities has continued. Researchers and educators alike have continued to address the pedagogic task of supporting learners to engage in literacy practices of production: a task made all the more challenging given the rapid rate of digital technology innovation and the constant emergence of new tools for design. Students need to know how to use the available digital media tools to access, produce and circulate texts in a dynamic communications environment. For example, as Burn and Durran (2007) argue, student control over the representational resources tied to software and hardware is an essential aspect of digital text composition. Production always involves choices about the medium of distribution, the networks through which to share, and many others, which form ‘part of what makes the text mean what it does and can affect the process of textual production’ (p. 19). A lack of technical knowledge about ‘how digital texts work’, on the part of teacher or student, can make digital literacy projects more complicated and time consuming in comparison to a writing project (DePalma & Alexander, 2015; Sheppard, 2009). At the same time, a number of models have been proposed to support educators in this area.

The development of visual grammar and design as conceptual frames for textuality has informed teachers’ approaches to digital and non-digital production. The visual grammar partially developed in the Australian Curriculum is informed by Kress and van Leeuwen’s (2006) visual grammar, which uses a systemic functional approach based on the linguistic work of Halliday (Halliday, 1978). In this context, a grammar is understood as a central system for organising ‘all the meaning-making resources in a mode’ (Collerson, 1997, p.2) and as a suitable overarching term for describing the regularities of a particular mode (Kress, 2003, p.65). A grammar provides users with a shared and systematic way to organise and talk about different meaning-making systems and the available semiotic resources. At the same time, grammars should not be seen as rigid and prescriptive (Halliday, 1978; Kress, 2003; Kress & van Leeuwen, 2006).

Classroom focused work exploring teaching visual design drawing on Kress and van Leeuwen’s (2006) visual grammar of still image (Callow, 2006; Edwards-Groves, 2011; Zammit, 2007) has expanded to include moving image (Buckingham & Willett, 2009). Research in this area shows the need for well-conceptualised models and a viable shared metalanguage for teaching and assessing meaning-making (Chandler, 2017; Cloonan 2011; Edwards-Groves, 2011; Unsworth, 2010).

Some of this need has been met by models for learning such as that advanced by multiliteracies learning by design pedagogy (Kalantzis & Cope, 2012; New London Group, 1996). Through these we can see how conceptual advances in semiotics and literacy education have become entangled with the conceptual evolution of digital literacy, again raising questions about the requisite knowledge and skills for those engaged in digital literacy education.

One conclusion that we offer from our research work navigating these entanglements (Bacalja, 2021; Beavis, 2022; Chandler, Unsworth and O'Brien, 2012) is that as others have noted (Bulfin & McGraw, 2015) skills-based approaches alone are insufficient if the goal is for young people to leave schooling as creative *and* critical users of digital technology, and an understanding about the ways their digital practices are tied to other cultural, social and economic conditions. Teaching orientated towards critical digital literacies offers one way to move practice beyond production. As the London School of Economics establish in their description of critical digital literacy, it is:

not only about evaluating online content but also understanding the internet’s production and consumption processes, its democratising potential, and its structural constraints. Critical digital literacy should be understood as essential in protecting people of all ages from misinformation, as well as in fostering social inclusion and the civic and political empowerment that comes from engaging reflexively with digital media…In order to be digital, critical digital literacy needs to incorporate understandings of the internet and the digital environment. (London LSE, 2015).

Given that the digital world will continue to evolve, whether we like it or not, digital literacy education also needs to progress, albeit in ways that might initially be uncomfortable for some.

### Data literacies for the digitally literate citizen

It is not enough for teachers to enact a version of digital literacy pedagogies that are limited to supporting students to design through digital media. We need to expand digital literacies to include data literacies. The rapid expansion of digital technologies into every aspect of contemporary life has contributed to what Barrasi calls ‘the datafication of everyday life’ (2018, p.170). Well beyond the use of apps to regulate a wide range of services that were once offered ‘in-person’, the intrusion of the digital now includes ‘the datafication of childhood’ (p.171), where children become datafied citizens as they are coerced into participating in society through digital means which leave digital traces. Lupton and Williamson (2017) refer to these young people as the ‘datafied child’, arguing that formal education systems are at the heart of monitoring and collecting data about learners as they move through schooling. The datafication of contemporary society does not stop at the school gates. As Williamson puts it, ‘A vast apparatus of measurement is being developed to underpin national educational systems, institutions and the actions of the individuals who occupy them’ (2017a, p.4). We should be concerned that the mass proliferation of digital devices, codes, software, and algorithms in education has gone largely unnoticed and unproblematised (Williamson, 2017a, 2017b). A brief look at two examples of the influence of the digital in schools, platformatisation and edu-apps, helps focus our attention on what a reconceptualised digital literacy education might constitute.

Platformatisation is the process of using digital platforms to offer digital services and encourage online participation. In the context of formal schooling, concerns have been raised about the extent to which such platforms shroud what they do with data collected about users’ activities. Kumar et al.’s (2019) investigation of teachers’ use of technology platforms found that the platformatisation of the classroom promoted surveillance and positioned teachers as surveillant consumers, gathering information for use in future decision making. Kerssens and Van Dijck (2021) exploration of the platformatisation of primary education in The Netherlands reported similar concerns about the impact of the increasing presence of platforms and data-driven services on classrooms. The integration of these digital systems into schools has been found to do more than just offer choices. They also impact pedagogical principles, social practices, and student interaction (Beetham & Sharpe, 2019). Pangrazio and Sefton-Green’s (2019) interest in how such platforms shape relationships for teaching and learning is particularly relevant for educators. They demonstrate how platforms form and inform relationships of learning. It is a reminder for those who believe teaching work is relational work, that technologies that interfere with such work also have the capacity to interfere with relationships, and that there is value in considering what knowledge students and teachers alike need if they are to be critically and digitally literate in their participation with these platforms inside and outside of schooling.

Another example of educational technology reshaping the digital literacies of schooling is the ubiquitous edu-app. While critical approaches to the study of educational apps have received only modest attention (Decuypere, 2019), the many ways these apps reorient the literacy practices of formal schooling require consideration. Decuypere’s (2019) analysis of one app, Grasshopper, an app designed to teach learners to code, challenges any notion of apps as neutral devices. As Decuypere explains, these apps ‘attempt to make students think in particular desired ways, seek to understand themselves and the world in certain predefined manners, and consequentially inscribe particular visions about education and learning and what it means to learn and be a learner today’ (p.3). Rennie et al.’s (2019) study of privacy and app use in Australian primary schools also demonstrates how important it is that professional learning programmes include an emphasis on digital technologies designed for school contexts. Rennie et al. found that privacy considerations were inconsistently apparent, and that the cost of apps often led to free apps being implemented despite the problematic issues around targeted advertising and the collection and selling of data from within the apps. They suggest that there is room for a more critically rigorous evaluation of privacy when it comes to selecting and using apps in classroom settings. They argue that when it comes to educational technology, Internet governance plays out in unique ways that are sensitive to the dispositions of individual teachers and schools.

We offer these examples as catalysts for asking questions about what teachers and students need to know, and be able to do, in response to the widespread incorporation of such technologies into formal schooling. Operationalising these technologies does not appear to be a challenge for most users. For the most part, platforms and apps are designed in ways that make their operation almost seamless. However, the risk is that this only contributes to their uncritical use. Approaches to digital literacy education that are limited to pedagogies supporting students to be digital makers and designers are not enough.

Importantly, work is already underway which considers digital citizenship in light of the issues caused by the expansion of digital literacies into almost every aspect of contemporary life. Emejulu and McGregor (2019) articulation of a ‘radical digital citizenship’, where ‘individuals and groups committed to social justice critically analyse the social, political, and economic consequences of digital technologies in everyday life and collectively deliberate and take action to build alternative and emancipatory technologies and technological practices’ (p.140), offers one set of approaches to helping teachers rethink the kinds of digital literacy practice in their classrooms. The centring of principles of justice, ethics and power represent a marked shift from skills-centric approaches. Similarly, Pangrazio and Selwyn’s (2021) case for ‘critical data education’ promotes the need for children to be taught how their data are collected and used, and what they can do to take control over their personal data. But multiple case studies undertaken by Pangrazio and Selwyn seeking to achieve such goals found that shifting young people’s digital practices was difficult given how deeply intertwined these are with ‘contemporary socialities’ (p.445). If the aim is for young and old alike to make more critically informed decisions about their participation in the digital world, then we should be prepared for a long and complex journey.

## Conclusion

This paper raises core questions about what constitutes digital literacy education. What, if anything, is the relationship between using digital texts and possessing the knowledge to critique such texts? Should students be aware that they are generating data as they use edu-apps and social media, and of the ways in which platforms and technologies make use of these? Should data literacy be included as part of critical digital literacy conceptualised as an expansion of traditional literacy terms? What are the implications for pedagogy and curriculum?

Some of the issues we raise reprise longstanding debates within the field. One set concerns conceptions of curriculum, and of the organisation of school learning, and what Green calls ‘the representation problem’, led by questions: what knowledge is of most worth? What should the schools teach? (Green, 2018, p.21).

Another issue relates to the great need for teachers to have access to specific knowledge about digital technologies, both hardware and software, and their features, and about how they might teach and work with them. Building knowledge in this field, and providing preservice and practising teachers with information, strategies and resources is patently a clear task for literacy educators, as for the profession as a whole.

As always, actions and possibilities are complex, often contradictory. Digital technologies and communication have massive roles, amongst them disseminating information and misinformation on the one hand, and enabling social cohesion and creative forms of meeting and relationships on the other; threatening or supporting mental health; facilitating workplaces and practices of some kinds, while limiting and risking others. One thing is sure. Now, more than ever, young people — and all of us — need to be critically and digitally literate, astute, and informed.
